# The utility of cardiopulmonary exercise testing in athletes and physically active individuals with or without persistent symptoms after COVID-19

**DOI:** 10.3389/fmed.2023.1128414

**Published:** 2023-04-26

**Authors:** Gisele Mendes Brito, Danilo Marcelo Leite do Prado, Diego Augusto Rezende, Luciana Diniz Nagem Janot de Matos, Irineu Loturco, Marcelo Luiz Campos Vieira, Ana Lúcia de Sá Pinto, Rodrigo Otávio Bougleux Alô, Lorena Christine Araújo de Albuquerque, Flavia Riva Bianchini, Ana Jéssica Pinto, Hamilton Roschel, Ítalo Ribeiro Lemes, Bruno Gualano, Coalition SPORT-COVID-19

**Affiliations:** ^1^Applied Physiology and Nutrition Research Group, School of Physical Education and Sport, and School of Medicine, University of São Paulo, São Paulo, Brazil; ^2^Hospital Israelita Albert Einstein, São Paulo, Brazil; ^3^Nucleous of High Performance in Sport, São Paulo, Brazil; ^4^Universidade Federal de São Paulo, São Paulo, Brazil; ^5^Instituto Dante Pazzanese de Cardiologia, São Paulo, Brazil; ^6^HCor Hospital do Coração, São Paulo, Brazil; ^7^Division of Endocrinology, Metabolism, and Diabetes and Anschutz Health and Wellness Center, University of Colorado Anschutz Medical Campus, Aurora, CO, United States

**Keywords:** SARS-CoV-2, sport, cardiorespiratory fitness, physical activity, recovery

## Abstract

**Introduction:**

Cardiopulmonary exercise testing (CPET) may capture potential impacts of COVID-19 during exercise. We described CPET data on athletes and physically active individuals with or without cardiorespiratory persistent symptoms.

**Methods:**

Participants’ assessment included medical history and physical examination, cardiac troponin T, resting electrocardiogram, spirometry and CPET. Persistent symptoms were defined as fatigue, dyspnea, chest pain, dizziness, tachycardia, and exertional intolerance persisting >2 months after COVID-19 diagnosis.

**Results:**

A total of 46 participants were included; sixteen (34.8%) were asymptomatic and thirty participants (65.2%) reported persistent symptoms, with fatigue and dyspnea being the most reported ones (43.5 and 28.1%). There were a higher proportion of symptomatic participants with abnormal data for slope of pulmonary ventilation to carbon dioxide production (VE/VCO_2_ slope; *p*<0.001), end-tidal carbon dioxide pressure at rest (PETCO2 rest; *p*=0.007), PETCO2 max (*p*=0.009), and dysfunctional breathing (*p*=0.023) vs. asymptomatic ones. Rates of abnormalities in other CPET variables were comparable between asymptomatic and symptomatic participants. When assessing only elite and highly trained athletes, differences in the rate of abnormal findings between asymptomatic and symptomatic participants were no longer statistically significant, except for expiratory air flow-to-percent of tidal volume ratio (EFL/VT) (more frequent among asymptomatic participants) and dysfunctional breathing (*p*=0.008).

**Discussion:**

A considerable proportion of consecutive athletes and physically active individuals presented with abnormalities on CPET after COVID-19, even those who had had no persistent cardiorespiratory symptomatology. However, the lack of control parameters (e.g., pre-infection data) or reference values for athletic populations preclude stablishing the causality between COVID-19 infection and CPET abnormalities as well as the clinical significance of these findings.

## 1. Introduction

The vast majority of athletes (i.e., 94%, including elite, collegiate, and amateurs) with COVID-19 are asymptomatic or exhibit mild acute symptoms (1). However, 8.3% may present with a broad range of post-acute symptoms, which may affect return-to-play decisions and timing (1).

Cardiopulmonary exercise testing (CPET) has been considered as a useful tool to assess cardiorespiratory fitness and its interplay with pathophysiologic and clinical manifestations in COVID-19 infected individuals. For individuals regularly engaged in exercise, CPET is thought to be particularly informative as it captures potential impacts of COVID-19 (e.g., diminished aerobic capacity and ventilatory inefficiency) that only manifest, or manifest more profoundly, during physical exertion (2). In fact, COVID-19 can affect lung and heart function, which may result in lower peak oxygen uptake, lower peak heart rate and stroke volume, and reduced ability to perform maximal exercise during CPET.

CPET has been primarily referred to individuals with post-COVID-19 symptomology, such as exertional dyspnea (3, 4). In a small cohort of athletes (*n* = 21) with persistent symptoms >28 days after COVID-19 diagnosis, CPET reproduced presenting symptoms in 86% of them. Moreover, improvements in symptomatology over time were accompanied by improvements in CPET variables (e.g., VO_2peak_, oxygen pulse, and resting and peak heart rate) (2). Importantly, even in the absence of any persistent symptoms, athletes can still experience subtle changes in lung function (e.g., dysfunctional breathing) and exercise capacity (e.g., deconditioning), which might be performance debilitating. Thus, these potential changes need to be detected and addressed to ensure a successful and safe return to sports. Nonetheless, the potential role of CPET in identifying subclinical, abnormal findings during physical exertion in exercised individuals without persistent symptomatology remains to be determined.

Herein we performed CPET on consecutive elite and highly trained athletes and physically active individuals with or without cardiopulmonary persistent symptoms. Our working hypotheses were that (i) those with symptomatology would show more abnormal findings during physical exertion, but (ii) CPET would be also able to identify abnormal spirometry in some athletes without any cardiopulmonary symptoms.

## 2. Materials and methods

This cross-sectional study is part of the Sport-COVID-19 Coalition and aimed to assess the health impacts of COVID-19 in elite and highly trained athletes and physically active individuals. The protocol was approved by the National and Institutional Ethical Committee of Hospital das Clínicas HCFMUSP, CAAE: 39260620.7.0000.0068. Written informed consent was obtained before participants’ enrollment.

Elite and highly trained athletes and physically active participants aged ≥18 years and diagnosed with COVID-19, either by IgG/IgM or RT-PCR, between 14 days and 36 weeks before the time of evaluation were eligible. Elite and highly trained athletes were defined as those performing structured and/or periodized training and are developing proficiency in skills required to perform their sport at the highest level (5). Physically active participants were defined as those meeting the World Health Organization minimum activity guidelines (6) or participating in multiple sports/forms of activity (5). Data collection was conducted between March 2021 and February 2022, during the predominance of delta and omicron variants in Brazil.

All participants underwent a thorough assessment including history and physical examination, cardiac troponin T, resting electrocardiogram (ECG), spirometry, and CPET. Cardiopulmonary persistent symptoms were defined as fatigue, dyspnea, chest pain, dizziness, tachycardia, and exertional intolerance persisting >2 months after COVID-19 diagnosis. Participants reporting at least one of these symptoms were defined hereafter as “symptomatic,” whereas those not reporting such symptoms were defined as “asymptomatic.” The detailed persistent symptomatology (including non-cardiopulmonary symptoms) was recorded through a survey conducted by a physician. All asymptomatic participants were actively training at the time of assessments. One symptomatic participant from the physically active group reported to be training at lower volume/intensity, whereas all others reported to be training normally.

CPETs were performed on a treadmill by the same exercise physiologist, with an intensity-graded, maximal effort protocol and continuous gas exchange (Metalyzer IIIb/breath-by-breath). For men, the test started at 5 km·h^−1^ and increased speed (1 km·h^−1^·min^−1^) up to a maximum velocity of 14 km·h^−1^. For women, the test started at 4 km·h^−1^ and increased speed (1 km·h^−1^·min^−1^) up to 13 km·h^−1^. For those participants who reached these maximal speeds, there was a subsequent increase in inclination (2%·min^−1^) until exhaustion. Ventilatory and gas exchange measurements were recorded continuously throughout the test using a breath-by-breath system (MetaLyzer 3B, Cortex), as was heart rate (HR; ergo PC elite, Micromed). Maximal effort was determined according to published criteria (7) and individual peak oxygen uptake was determined as the VO_2_ averaged over the final 30 s. The ventilatory anaerobic threshold (VAT) was determined to occur at the breakpoint between the increase in the carbon dioxide output and VO_2_, or at the point at which the minute ventilation/carbon dioxide production (VE/VO_2_) reached a minimum value and began to rise without a concomitant rise in the ventilatory equivalent for carbon dioxide (VE/VCO_2_) (8). The respiratory compensation point (RCP) was determined to be the point at which the VE/VCO_2_ reached a minimum value and began to rise, and the carbon dioxide partial pressure (PETCO_2_) reached its highest value before its progressive fall. Test termination was determined by volitional exhaustion and maximal effort was confirmed by a peak respiratory exchange ratio ≥ 1.10, maximal heart rate > 95% age/gender-predicted values, or maximum rating of perceived exertion (RPE) (9).

The pulmonary function test was conducted before and 6 min after CPET according to established recommendations (ATS-spirometry) (9). Forced vital capacity (FVC) (L), forced expiratory volume in the first second (FEV1) (L), FEV1/FVC (%), and peak expiratory flow (PEF) (L/s) were evaluated.

The outcomes were absolute and % predicted values of oxygen consumption at ventilatory anaerobic threshold (VO_2VAT_) (ml/kg/min), VO_2peak_ (ml/kg/min), and oxygen uptake efficiency slope (OUES) (L/min).

To assess ventilatory limitation and efficiency, we measured breathing reserve [peak pulmonary ventilation-to-maximum voluntary ventilation ratio (VE/MVV)], slope of pulmonary ventilation to carbon dioxide production from the beginning of exercise to the respiratory compensation point (VE/VCO_2_ rest-RCP), and the highest value for end-tidal carbon dioxide pressure (PETCO_2_ Max) (mmHg). To assess the degree of the expiratory flow limitation during CPET, we used tidal flow-volume loop obtained in the last 20 s for each workload and plotted it within the maximal FVC obtained at rest. We also evaluated expiratory air flow-to-percent of tidal volume (EFL/VT) ratio (%) that meets or exceeds the expiratory boundary of the FVC. To assess cardiocirculatory efficiency, we measured O_2_ pulse (ml/beat), cardiocirculatory efficiency [heart rate-to-VO_2_ ratio (HR/VO_2_)] (bpm/L/min), and HR recovery 1 min after exercise (HRR-1) (%). The running economy was calculated at intensities corresponding to the ventilatory anaerobic threshold (RE_VAT_) and respiratory compensation point (RE_RCP_) and were calculated as the oxygen cost to cover a given distance using the following equation: (10)


$$\begin{array}{l} \text{RE}\;(\text{ml}\;O2.\text{kg}-1.\text{km}-1)=\text{VO}2\;(\text{ml}.\text{kg}-1.h-1)\;\\ \;x\;60/\text{speed}\;(\text{Km}.h-1) \end{array}$$

Abnormal findings were defined as those deviating from normality values, according to well-accepted references (9, 11–15). Dysfunctional breathing was determined by the presence of one or more of the following features: high VE/VCO_2_ (>35 during exercise), low PETCO_2_ (< 4 kPa both at rest and during exercise), or erratic tidal volume and/or respiratory rate (RR) response to workload (16). Deconditioning was determined by the presence of one or more of the following features: reduced VO_2peak_, reduced slope or late plateau of theVO_2_ response (i.e., VO_2_/work-rate relationship ≤8), or a premature anaerobic threshold (i.e., <40% predicted) (13).

The number of participants was chosen based on feasibility, capacity of research staff and facility, resources, and available participants, in accordance with previous recommendations (17).

Descriptive statistics were used to summarize participants’ characteristics. Continuous variables were described as means and standard deviation (SD), and categorical variables were presented as absolute and percentage values. Differences between asymptomatic and symptomatic participants’ demographic characteristics, CPET and spirometry results were tested using Student’s T-tests for continuous variables, and Chi-square tests for categorical variables. The relationship between cardiopulmonary persistent symptoms (yes/no) and normality values of CPET and spirometry variables were assessed by Chi-square test. A sub-analysis was performed including only elite and highly trained athletes (*n* = 26). Analyses were conducted using SPSS (version 26.0, SPSS Inc., Chicago, IL, USA) and statistical significance (value of *p*) was set at 0.05.

## 3. Results

A total of 26 elite/highly trained athletes and 20 physically active participants were included. Mean age was 30 ± 9 yrs., body mass index (BMI) was 25.6 ± 5.2 kg/m^2^, and training volume was 14.8 ± 6.5 h/week. Thirty participants (65.2%) reported having persistent COVID-19 symptoms, with fatigue and dyspnea being the most reported symptoms (43.5% and 28.1%, respectively). On average, CPET was performed 14.4 ± 8.5 weeks after COVID-19 diagnosis. Table 1 depicts the main participants’ characteristics. The most frequent abnormal findings in CPET and spirometry variables were VE/VCO_2_ slope, PETCO2 rest (mmHg) and PEF (bpm/L/min) among symptomatic participants, and PEF (bpm/L/min), EFL/VT (%) and ECG alterations (i.e., altered rhythm, ectopic foci, or ST segment changes during CPET and/or recovery that did not lead to test termination) among asymptomatic participants. Dysfunctional breathing was more frequent among symptomatic participants (53.3% vs. 18.8%) (Table 2). Three participants had altered cardiac troponin T levels. None of them had dynamic ECG alterations; yet, we decided to refer them to echocardiogram. Two had normal exams, whereas one did not attend the assessment. All CPET were interrupted because of leg fatigue. No one reported dyspnea as a cause for stopping exercising.

**Table 1:** Participants’ characteristics.

Participant’s characteristics	Total sample (*n* = 46)	Asymptomatic (*n* = 16)	Symptomatic (*n* = 30)	Value of *p*^c^
Age (years)	30 ± 9	27 ± 9	32 ± 9	0.102
Sex [female, *n* (%)]	20 (43.5)	2 (12.5)	18 (60.0)	0.002
BMI (kg/m^2^)	25.6 ± 5.2	28.0 ± 6.0	24.3 ± 4.3	0.039
Training status				
Training volume (hours/week)	14.8 ± 6.5	15.3 ± 4.6	14.6 ± 7.4	0.226
Modality				
Soccer	8 (17.4)	5 (31.3)	3 (10.0)	0.003
Crossfit®	6 (13.0)	0 (0.0)	6 (20.0)	
Rugby	5 (10.9)	4 (25.0)	1 (3.3)	
Triathlon	4 (8.7)	0 (0.0)	4 (13.3)	
Para Athletics	3 (6.5)	1 (6.3)	2 (6.7)	
Judo	3 (6.5)	2 (12.5)	1 (3.3)	
Taekwondo	3 (6.5)	2 (12.5)	1 (3.3)	
Squash	3 (6.5)	0 (0.0)	3 (10.0)	
Running	2 (4.3)	0 (0.0)	2 (6.7)	
Other^a^	9 (19.6)	2 (12.5)	7 (23.3)	
Classification				
Elite/Highly trained athletes	26 (56.5)	15 (88.2)	11 (36.7)	<0.001
Physically active	20 (43.5)	1 (11.8)	19 (63.3)	
Vaccinated^b^ [yes, *n* (%)]	18 (39.1)	4 (25)	14 (47)	0.220
Acute COVID-19 symptomatology [*n* (%)]				
Asymptomatic	4 (8.7)	2 (12.5)	2 (6.7)	0.778
Mild	35 (76.1)	12 (75.0)	23 (76.7)	
Moderate	5 (10.9)	1 (6.3)	4 (13.3)	
Severe	2 (4.3)	1 (6.3)	1 (3.3)	
Persistent COVID-19 symptoms [*n* (%)]	30 (65.2)	N/A	30 (100.0)	N/A
Dyspnea	14 (30.4)	N/A	14 (46.7)	
Fatigue	19 (41.3)	N/A	19 (63.3)	
Chest discomfort	6 (13.0)	N/A	6 (20.0)	
Tachycardia	3 (6.5)	N/A	3 (10.0)	
Musculoskeletal pain	4 (8.7)	N/A	4 (13.3)	
Headache	8 (17.4)	N/A	8 (26.7)	
Dizziness	1 (2.2)	N/A	1 (3.3)	
Diarrhea	1 (2.2)	N/A	1 (3.3)	

Data is reported as mean ± SD or N (%). BMI, body mass index; N/A, not applicable.

a Swimming, cycling, volleyball, pilates, strongman, rowing, karate, futsal, athletics.

b At the time of infection.

**Table 2:** CPET and spirometry variables according to the presence of cardiopulmonary persistent symptoms.

CPET and spirometry variables	Total sample (*n* = 46)	Asymptomatic (*n* = 16)	Symptomatic (*n* = 30)	Value of *p*^c^
VO_2VAT_ (ml/kg/min)	23.7 ± 6.3	24.2 ± 6.7	23.5 ± 6.2	0.718
VO_2VAT_ (% predicted)	68.5 ± 16.7	65.9 ± 12.2	69.8 ± 18.7	0.398
VO_2peak_ (ml/kg/min)	41.1 ± 8.7	41.9 ± 8.3	40.6 ± 9.1	0.627
VO_2peak_ (% predicted)	118.0 ± 18.8	114.9 ± 9.7	119.7 ± 22.2	0.320
HR_max_ (bpm)	180.1 ± 9.4	181.1 ± 8.8	179.6 ± 9.7	0.608
RER_peak_	1.1 ± 0.1	1.2 ± 0.1	1.1 ± 0.1	0.022
OUES (L/min)	3.2 ± 0.8	3.7 ± 0.7	2.9 ± 0.8	0.002
VE/MVV peak	31.9 ± 23.2	29.1 ± 20.3	33.4 ± 24.8	0.536
VE/VCO_2_ slope	29.7 ± 3.1	28.0 ± 1.2	30.6 ± 3.5	0.001
PETCO_2_ rest (mmHg)	31.4 ± 3.9	33.8 ± 3.1	30.1 ± 3.7	0.001
PETCO_2_ max (mmHg)	37.2 ± 3.4	39.4 ± 2.2	36.0 ± 3.3	<0.001
Predicted O_2_ pulse (%)	113.4 ± 20.1	112.0 ± 9.8	114.1 ± 24.0	0.676
FVC (L)	4.6 ± 1.3	5.4 ± 1.3	4.1 ± 1.1	0.003
FEV1 (L)	4.1 ± 1.2	4.8 ± 1.2	3.7 ± 1.0	0.005
FEV1/FVC (%)	90.5 + 7.5	89.3 ± 8.2	91.1 ± 7.1	0.476
PEF (L/s)	8.6 ± 2.7	9.5 ± 3.0	8.2 ± 2.5	0.148
HR/VO_2_ (bpm/L/min)	40.0 ± 13.5	32.9 ± 8.8	43.7 ± 14.2	0.003
HRR-1 (bpm)	16.8 ± 8.4	14.8 ± 7.8	17.9 ± 8.7	0.223
EFL/VT (%)	13.9 ± 29.9	22.6 ± 28.6	9.2 ± 21.7	0.114
RE_VAT_ (ml/kg/km)	188.2 ± 32.2	186.9 ± 33.2	188.9 ± 32.1	0.848
RE_RCP_ (ml/kg/km)	173.6 ± 20.8	169.6 ± 18.7	175.7 ± 21.9	0.330
Dysfunctional breathing [*n* (%)]^d^	19 (41.3)	3 (18.8)	16 (53.3)	0.023
Deconditioning [*n* (%)]^e^	9 (19.6)	2 (12.5)	7 (23.3)	0.378
ECG alterations^a^ [*n* (%)]	18 (39.1)	9 (52.9)	9 (30.0)	0.082
Isolated ventricular ectopic foci	13 (28.3)	7 (43,7)	6 (20.0)	0.215
Supraventricular ectopic foci	4 (8.7)	2 (12.5)	2 (6.7)	
Bigeminated rhythm	2 (4.3)	2 (12.5)	0 (0.0)	
Ventricular repolarization change	2 (4.3)	0 (0.0)	2 (6.7)	
Abnormal Troponin T^b^	3 (7.1)	1 (7.1)	2 (7.1)	0.209

Data is reported as mean ± SD or N (%). CPET, cardiopulmonary testing; RER, respiratory exchange ratio; VO_2VAT_, oxygen consumption at ventilatory anaerobic threshold; VO_2peak_, peak oxygen consumption; OUES, oxygen uptake efficiency slope; VE, ventilatory equivalent; MVV, maximum voluntary ventilation; VCO_2_, carbon dioxide output; PETCO_2_, patient end-tidal carbon dioxide; FVC, forced vital capacity; FEV1, forced expiratory volume in the first second; PEF, peak expiratory flow; HR, heart rate; HRR-1, heart rate recovery 1 min after exercise; EFL, expiratory air flow; VT, tidal volume; RE_VAT_, running economy at ventilatory anaerobic threshold; RE_RCP_, running economy at respiratory compensation point; ECG, electrocardiogram; N/A, not applicable.

a Altered rhythm, ectopic foci, or ST segment changes during CPET and/or recovery that did not lead to test termination (18).

b Missing data: 4 participants (asymptomatic, *n* = 2; symptomatic, *n* = 2).

c Asymptomatic vs. symptomatic.

d Defined as the presence of one or more of these features: high VE/VCO_2_ (>35 during exercise), low PETCO2 (< 4 kPa both at rest and during exercise), and erratic tidal volume and/or respiratory rate (RR) response to workload.

e Defined as the presence of one or more of these features: reduced VO_2peak_, reduced slope or late plateau of theVO2 response (i.e., VO2/work-rate relationship ≤ 8) and a premature anaerobic threshold (i.e., <40% predicted).

There was a higher proportion of symptomatic participants with abnormal data for VE/VCO_2_ slope (*p* < 0.001), PETCO_2_ rest (*p* = 0.007), and PETCO_2_ max (*p* = 0.009) vs. asymptomatic participants. In contrast, abnormalities in EFL/VT were more frequent among asymptomatic participants (*p* = 0.012). Rate of abnormalities in other CPET variables was comparable between asymptomatic and symptomatic participants (Table 3).

**Table 3:** Prevalence of abnormal findings on cardiopulmonary exercise test and spirometry variables according to the presence of persistent symptoms after COVID-19.

Variable	Expected values	Asymptomatic	Symptomatic	*p-*value
		Abnormal findings *n* (%)	Abnormal findings *n* (%)	
Total sample (*n* = 46)		*n* = 16	*n* = 30	
VO_2VAT_ (% predicted)	>40%^9^	0 (0.0)	3 (10.0)	0.191
VO_2peak_ (% predicted)	>84%^9^	0 (0.0)	2 (6.7)	0.291
OUES (L/min)	>1.05^12^	0 (0.0)	0 (0.0)	N/A
VE/MVV peak	>20%^13^	5 (29.4)	11 (36.7)	0.713
VE/VCO_2_ slope	≤30^23^	1 (5.9)	19 (63.3)	<0.001
PETCO_2_ rest (mmHg)	>33^12^	4 (23.5)	20 (66.7)	0.007
PETCO_2_ max (mmHg)	>36^12^	0 (0.0)	10 (33.3)	0.009
Predicted O_2_ pulse (%)	>80%^9^	0 (0.0)	1 (3.2)	0.460
FVC (L)	>3.1^14^	4 (23.5)	9 (30.0)	0.720
FEV1 (L)	>2.6^14^	1 (5.9)	6 (20.0)	0.216
FEV1/FVC (%)	>81%^14^	2 (11.8)	1 (3.3)	0.230
PEF (L/s)	7.1–11.1^14^	11 (64.7)	19 (63.3)	0.713
HR/VO_2_ (bpm/L/min)	≤50^13^	1 (5.9)	7 (23.3)	0.145
HRR-1 (bpm)	>12^12^	6 (35.3)	8 (26.7)	0.447
EFL/VT (%)	<25%^13^	9 (52.9)	6 (20.0)	0.012
RE_VAT_ (ml/kg/km)	<218^15^	2 (11.8)	4 (13.3)	0.936
RE_RCP_ (ml/kg/km)	<218^15^	0 (0.0)	2 (6.7)	0.291
Dysfunctional breathing [*n* (%)]^c^		3 (18.8)	16 (53.3)	0.023
Deconditioning [*n* (%)]^d^		2 (12.5)	7 (23.3)	0.378
ECG alterations^a^ [*n* (%)]		9 (52.9)	9 (30.0)	0.082
Elite/Highly trained athletes (*n* = 26)		*n* = 15	*n* = 11	
VO_2VAT_ (% predicted)		0 (0.0)	1 (9.1)	0.234
VO_2peak_ (% predicted)		0 (0.0)	1 (9.1)	0.234
OUES (L/min)		0 (0.0)	0 (0.0)	N/A
VE/MVV peak		5 (33.3)	4 (36.4)	0.873
VE/VCO_2_ slope		1 (6.7)	4 (36.4)	0.058
PETCO_2_ rest (mmHg)		4 (26.7)	7 (63.6)	0.059
PETCO_2_ max (mmHg)		0 (0.0)	2 (18.2)	0.086
Predicted O_2_ pulse (%)		0 (0.0)	0 (0.0)	N/A
FVC (L)		4 (26.7)	3 (27.3)	0.973
FEV1 (L)		1 (6.7)	2 (18.2)	0.364
FEV1/FVC (%)		2 (13.3)	0 (0.0)	0.207
PEF (L/s)		11 (73.3)	7 (63.6)	0.597
HR/VO_2_ (bpm/L/min)		1 (6.7)	0 (0.0)	0.382
HHR-1 (bpm)		5 (33.3)	4 (36.4)	0.873
EFL/VT (%)		9 (60.0)	2 (18.2)	0.033
RE_VAT_ (ml/kg/km)		2 (13.3)	2 (18.2)	0.735
RE_RCP_ (ml/kg/km)		0 (0.0)	2 (18.2)	0.086
Dysfunctional breathing [*n* (%)]		2 (13.3)	7 (63.6)	0.008
Deconditioning [*n* (%)]		2 (13.3)	2 (18.2)	0.735
ECG alterations^a^ [*n* (%)]		9 (60.0)	3 (27.3)	0.098

BMI, body mass index; CPET, cardiopulmonary testing; RER, respiratory exchange ratio; VO_2VAT_, oxygen consumption at ventilatory anaerobic threshold; VO_2peak_, peak oxygen consumption; OUES, oxygen uptake efficiency slope; VE, ventilatory equivalent; MVV, maximum voluntary ventilation; VCO_2_, carbon dioxide output; PETCO_2_, patient end-tidal carbon dioxide; FVC, forced vital capacity; FEV1, forced expiratory volume in the first second; PEF, peak expiratory flow; HR, heart rate; HRR-1, heart rate recovery 1 min after exercise; EFL, expiratory air flow; VT, tidal volume; RE_VAT_, running economy at ventilatory anaerobic threshold; RE_RCP_, running economy at respiratory compensation point; ECG, electrocardiogram; N/A, not applicable.

a Altered rhythm, ectopic foci, or ST segment changes during CPET and/or in recovery that did not lead to test termination (18).

c Defined as the presence of one or more of these features: high VE/VCO2 (>35 during exercise), low PETCO2 (<4 kPa both at rest and during exercise), and erratic tidal volume and/or respiratory rate (RR) response to workload.

d Defined as the presence of one or more of these features: reduced VO2peak, reduced slope or late plateau of the VO2 response (i.e., VO2/work-rate relationship ≤ 8) and a premature anaerobic threshold (i.e., <40% predicted).

Among elite and highly trained athletes, differences in the rate of abnormal findings between asymptomatic and symptomatic participants observed for the total sample were no longer statistically significant (all *p* > 0.050), except for EFL/VT, which were more frequent among asymptomatic participants (*p* = 0.033), and dysfunctional breathing, which were more frequent among symptomatic athletes (*p* = 0.008) (Table 3).

## 4. Discussion

**Figure 1:**
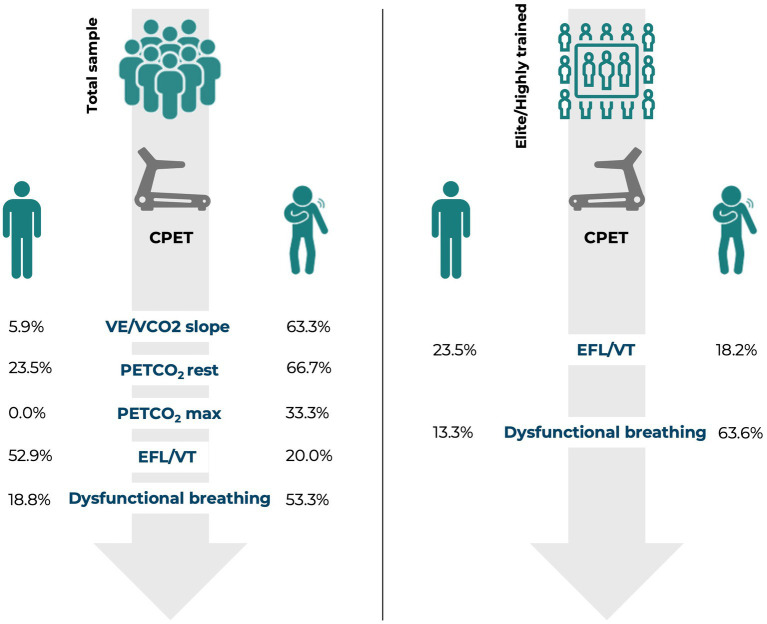
Proportion of asymptomatic vs. symptomatic participants with abnormal CPET findings.

This study described CPET data on consecutive athletes and physically active individuals as a function of persistent cardiorespiratory symptomatology. The key findings were threefold: (i) considering the overall sample, the rate of symptomatic participants (those with persistent cardiopulmonary symptoms) exhibiting abnormal CPET findings associated with diminished aerobic capacity and ventilatory inefficiency (e.g., VE/VCO_2_ slope) was higher than that of asymptomatic participants; (ii) CPET may also be useful to identify a few abnormal physiological responses (e.g., HRR-1, PEF) during and after exercise in some asymptomatic participants; and (iii) among elite and highly trained athletes, the proportion of CPET abnormalities were comparable between symptomatic and asymptomatic ones, and running economy was not affected (Figure 1). Overall, these findings are of clinical relevance as discussed thereafter.

Our findings concur with previous studies showing abnormalities in spirometry among a variety of symptomatic athletes (19–21). However, we also found a high proportion of participants exhibiting abnormal ventilatory efficiency, which is not frequently reported in other cohorts (2, 19–22). These studies are difficult to reconcile since the largely vary in terms of timing of CPET in relation to COVID-19 onset and the presence of persistent symptoms. Our sensitivity analysis comprising symptomatic elite and highly trained athletes showed a lower number of cases with abnormal values for VE/VCO_2_ slope (36.4%) vs. those found in the general sample (63.3%), which also included recreationally trained individuals. This suggests that training status may be a factor affecting ventilatory efficiency abnormalities following COVID-19, with highly trained individuals being likely less susceptible to impairments in ventilatory efficiency than less trained ones. The exact mechanisms underlying the increased VE/VCO_2_ slope among symptomatic patients are yet to be fully elucidated; however, it is postulated that residual lung impairment, deconditioning, persistent inflammatory response, and neuropsychological changes (e.g., anxiety, depressive symptoms) may play a role in this alteration. On the other hand, a low breathing reserve (as assessed by VE/MVV peak), for example, appeared to manifest regardless of training status. Indeed, how COVID-19 distinctly affects individuals with different training levels requires deeper investigation.

One interesting data of this study was the ability of CPET not only to reproduce persistent cardiorespiratory symptomatology in exercised individuals, but also to detect a few abnormalities in asymptomatic ones. For instance, participants without persistent symptoms showed high rates of alterations in PEF (~65%), EFL/VT (~53%), and HRR-1 (~35%). These findings suggest that even exercised, asymptomatic individuals may experience subclinical abnormalities in ventilatory responses (e.g., alterations in breathing mechanics, expiratory flow limitation, increased ventilatory demands) and slower post-exercise HR recovery, which is suggestive of dysautonomia (23), although it is impossible to determine whether these alterations are linked to COVID-19 or pre-existing.

Symptomatic individuals did have more abnormalities on CPET and spirometry than asymptomatic counterparts; however, when elite/highly trained athletes were analyzed separately, persistent symptomatology did not clearly associate with CPET findings, except for dysfunctional breathing. It has been suggested that CPET has a great clinical utility by identifying cardiorespiratory abnormalities that may serve as therapeutic targets in athletes presenting with a high burden of persistent or late-onset cardiopulmonary symptoms after COVID-19 (18). The present study extends this notion to athletes and physically active individuals who show no persistent symptomatology. Although the widespread referral of CPET and/or spirometry is obviously not feasible, it might be useful within elite sports, whenever available. The rationale would be that, while the CPET abnormalities observed herein do not seem to pose significative health-related risk to return to training or competitions, they may limit optimal exercise performance, which is particularly concerning for elite athletes, rather than recreationally exercised invididuals (1). For instance, some athletes presented with dysfunctional breathing (*n* = 9) and deconditioning (*n* = 4) on CPET, both being potentially performance-debilitating characteristics. New trials aimed at treating abnormal CPET and spirometry to abbreviate return to training and optimal performance recovery are necessary.

This study has several limitations. First, our cross-sectional design does not allow determining the course of CPET abnormalities. As abnormal data were judged by references values obtained by non-athletic populations, we were unable to confirm the clinical significance of the findings (i.e., the impacts on health and performance). Second, the sample was selected by convenience, and it does not represent the full spectrum of sports modalities and training status, nor is reflective of the usual low rates of symptomatic cases after acute infection (1). Third, we did not have CPET data before COVID-19 infection to confirm that the observed abnormalities were not pre-existing. Fourth, this study was carried out during the predominance of different variants, but the impact of each of them was not possible to be determined. Fifth, it was not feasible for us to measure operating lung volumes by serial inspiratory capacity maneuvers during exercise to assess ventilatory constraints and define ventilatory limitation during exertion. Nevertheless, we measured spontaneous operational lung volume and calculated the EFL/VT, which enabled us to evaluate expiratory flow limitation during exercise, being considered important for determining the etiology of dyspnea (24). Lastly, most participants had not been vaccinated before being infected in this study, so that the role of vaccination on post-COVID symptoms and CPET abnormalities remains to be addressed.

In conclusion, a considerable proportion of consecutive athletes and physically active individuals presented with abnormalities on CPET after COVID-19, even those who had had no persistent cardiorespiratory symptomatology. These findings reinforce the notion that CPET should be primarily referred to athletes/physically active individuals with persistent symptoms; whenever available, it could be also indicated for elite athletes irrespective of symptomatology, as a potential tool for assessing cardiorespiratory status, detect potential subclinical cardiorespiratory abnormalities, and inform optimal rehabilitative strategies for recovering. Importantly, the reference values used herein for indicating cardiorespiratory abnormalities derived from non-athletic populations, so that this study does not allow stablishing the actual clinical significance of the current findings. The causality of COVID-19 infection and abnormal CPET findings still remains to be proven by controlled studies.

## Data availability statement

The raw data supporting the conclusions of this article will be made available by the authors, without undue reservation.

## Ethics statement

The protocol was approved by the National and Institutional Ethical Committee of Hospital das Clínicas HCFMUSP, CAAE: 39260620.7.0000.0068. Written informed consent was obtained before participants’ enrollment.

## Coalition SPORT-COVID-19

Gilberto Scarf, Hospital Israelita Albert Einstein/Federal University of São Paulo; Nabil Ghorayeb; Bruno Bassaneze, HCor Hospital do Coração; Marcos Perillo Filho, Thiago Ghorayeb Garcia, Instituto Dante Pazzanese de Cardiologia; Mateus Freitas Teixeira, Clube de Regatas Vasco da Gama/Complexo Hospitalar de Niterói; Fernanda Rodrigues Lima, Marina Valente Guimarães Cecchini, University of São Paulo.

## Author contributions

GB and DP collected the data. GB, DP, LM, AP, HR, ÍL, and BG analyzed data and interpreted results. BG, AP, and ÍL drafted the manuscript. The Coalition SPORT-COVID conceived and designed the study, edited and revised the manuscript, and approved the final version. All authors contributed to the article and approved the submitted version.

## Funding

The authors are thankful to São Paulo Research Foundation (FAPESP; grants #2015/26937-4 #2017/13552-2, #2018/19418-9 and #2020/04877-8), and Conselho Nacional de Desenvolvimento Científico e Tecnológico (CNPq).

## Conflict of interest

The authors declare that the research was conducted in the absence of any commercial or financial relationships that could be construed as a potential conflict of interest.

## Publisher’s note

All claims expressed in this article are solely those of the authors and do not necessarily represent those of their affiliated organizations, or those of the publisher, the editors and the reviewers. Any product that may be evaluated in this article, or claim that may be made by its manufacturer, is not guaranteed or endorsed by the publisher.
